# Lipidic cubic phase serial millisecond crystallography using synchrotron radiation

**DOI:** 10.1107/S2052252514026487

**Published:** 2015-01-27

**Authors:** Przemyslaw Nogly, Daniel James, Dingjie Wang, Thomas A. White, Nadia Zatsepin, Anastasya Shilova, Garrett Nelson, Haiguang Liu, Linda Johansson, Michael Heymann, Kathrin Jaeger, Markus Metz, Cecilia Wickstrand, Wenting Wu, Petra Båth, Peter Berntsen, Dominik Oberthuer, Valerie Panneels, Vadim Cherezov, Henry Chapman, Gebhard Schertler, Richard Neutze, John Spence, Isabel Moraes, Manfred Burghammer, Joerg Standfuss, Uwe Weierstall

**Affiliations:** a Laboratory for Biomolecular Research, Paul Scherrer Institute, Villigen 5232, Switzerland; b Department of Physics, Arizona State University, Tempe, AZ 85287, USA; c Center for Free-Electron Laser Science, Deutsches Elektronen-Synchrotron DESY, Hamburg 22607, Germany; d European Synchrotron Radiation Facility, Grenoble Cedex 9, F-38043, France; e Department of Integrative Structural and Computational Biology, Scripps Research Institute, La Jolla, California USA; f Centre for Ultrafast Imaging, Hamburg 22607, Germany; g Department of Chemistry and Molecular Biology, University of Gothenburg, Gothenburg, Sweden; h Department of Physics, University of Hamburg, Hamburg 22607, Germany; i Deparment of Biology, ETH Zurich, Zürich 8093, Switzerland; j Membrane Protein Laboratory, Diamond Light Source, Harwell Science and Innovation Campus, Chilton, Oxfordshire OX11 0DE, England; k Department of Life Sciences, Imperial College London, London, England; l Research Complex at Harwell Rutherford, Appleton Laboratory, Harwell, Didcot, Oxfordshire OX11 0FA, England; m Department of Analytical Chemistry, Ghent University, Ghent B-9000, Belgium

**Keywords:** lipidic cubic phases, protein crystallography, bacteriorhodopsin, XFEL

## Abstract

Lipidic cubic phases (LCPs) have emerged as successful matrixes for the crystallization of membrane proteins. Moreover, the viscous LCP also provides a highly effective delivery medium for serial femtosecond crystallography (SFX) at X-ray free-electron lasers (XFELs). Here, the adaptation of this technology to perform serial millisecond crystallography (SMX) at more widely available synchrotron microfocus beamlines is described. Compared with conventional microcrystallography, LCP-SMX eliminates the need for difficult handling of individual crystals and allows for data collection at room temperature. The technology is demonstrated by solving a structure of the light-driven proton-pump bacteriorhodopsin (bR) at a resolution of 2.4 Å. The room-temperature structure of bR is very similar to previous cryogenic structures but shows small yet distinct differences in the retinal ligand and proton-transfer pathway.

## Abstract 2

This article describes the structure determination of a membrane protein by serial injection of microcrystals in lipidic cubic phases into a synchrotron microfocus beam. The method is discussed with respect to serial femtosecond crystallography at free-electron lasers.

## 1. Introduction   

Structure determination by X-ray crystallography has developed continuously over the last century, yielding structures of ever more difficult and complex molecules. An important development is synchrotron-based microcrystallography, which uses brilliant X-ray beams of a few micrometres in diameter to collect data from very small weakly diffracting crystals. Microcrystallography has matured over the last few years (Smith *et al.*, 2012 ▶), but structure determination using microcrystals remains challenging and radiation damage limits the achievable resolution for well ordered small crystals (Garman, 2010*a* ▶). Microcrystallography has been particularly successful with membrane proteins grown in lipidic cubic phases (LCP). Crystallization in LCP environments often produces crystals that are highly ordered but limited in size. Protein crystallization in LCP was introduced 18 years ago (Landau & Rosenbusch, 1996 ▶) and has proven crucial for determining high-resolution structures and functional mechanisms of membrane proteins from several families, such as microbial rhodopsins, G protein-coupled receptors, ion channels, transporters and enzymes (Cherezov, 2011 ▶).

Protein microcrystals grown in LCP are well suited for the emerging technique of serial femtosecond crystallography (SFX) at X-ray free-electron lasers (XFELs) (Chapman *et al.*, 2011 ▶; Fromme & Spence, 2011 ▶; Spence *et al.*, 2012 ▶), in which micro- and nanometre-sized protein crystals are injected across ultrafast X-ray pulses in a stream at room temperature. Due to the high flux density, each crystal is destroyed by the photoelectron cascade following the X-ray pulse, but the duration of each XFEL pulse is so brief (typically ∼40 fs) that it terminates before conventional types of radiation damage have manifested themselves (Neutze *et al.*, 2000 ▶). Therefore, only a single diffraction pattern per crystal, which contains information on essentially undamaged molecules, is collected. A gas dynamic virtual nozzle (GDVN) (DePonte *et al.*, 2008 ▶; Weierstall *et al.*, 2012 ▶), which was the injection device for these first experiments (Chapman *et al.*, 2011 ▶; Boutet *et al.*, 2012 ▶; Johansson *et al.*, 2012 ▶), can deliver crystals in their low-viscosity crystallization buffer/mother liquor at a liquid flow rate of about 10 µl min^−1^ and a speed of about 10 m s^−1^. At this flow rate, and with the repetition rate of the hard XFEL sources currently in operation, most crystals flow past the interaction point in the time between X-ray pulses and are therefore wasted. This results in a requirement of up to 100 mg of protein for a single complete data set, and obtaining such large amounts is not feasible for many membrane proteins. Due to its high viscosity, the LCP can be extruded at much lower stream speeds (1–300 nl min^−1^), but it is incompatible with the GDVN device. A newly developed LCP injector (Weierstall *et al.*, 2014 ▶) extrudes a 20–50 µm diameter stream of LCP into ambient air or vacuum. It reduces sample consumption 50–100 fold compared with the GDVN and has already been used to solve several membrane protein structures (Liu *et al.*, 2013 ▶; Caffrey *et al.*, 2014 ▶; Weierstall *et al.*, 2014 ▶) at the Linac Coherent Light Source (LCLS, Stanford, California, USA), the first hard X-ray FEL.

Recently, the structural biology community has begun to adopt serial approaches to structure determination at third-generation synchrotron sources. Gati *et al.* (2014 ▶) used helical line scans to solve the structure of *Trypanosoma brucei* procathepsin B from cryocooled crystals. The first serial crystallography experiment at a synchrotron yielded a 2.1 Å lysozyme structure by merging single frames from microcrystals injected randomly into the X-ray beam in a glass capillary (Stellato *et al.*, 2014 ▶). Our LCP windowless injector allows the stream velocity to be slowed down to a rate of 0.05–0.15 µm ms^−1^, which allows 10–100 ms exposure times and efficient use of the sample. Here, we demonstrate that this makes it possible to perform LCP microjet-based serial millisecond crystallography (SMX) using synchrotron radiation, similar to SFX at an XFEL. The key advantages of this method are: (i) crystal injection using the LCP combined with a microfocus beamline allows diffraction data to be collected at room temperature, and hence crystal freezing and difficult crystal handling steps such as mounting crystals in a loop are not necessary; (ii) thousands of crystals can be screened in a short time and with less than a milligram of protein; (iii) microfocus beams at storage-ring sources are widely available and hence beam access is unlikely to limit SMX; and (iv) the method is well suited for time-resolved diffraction studies on the microsecond to millisecond timescale.

## 2. Methods   

### 2.1. Purification   

Bacteriorhodopsin (bR) was purified from purple membranes of *Halobacterium salinarum* as described by Nollert (2004 ▶), with modifications. All steps were performed under dim red light or in the dark. The purple membrane was resuspended in 50 m*M* NaH_2_PO_4_ pH 6.9 (GERBU Biotechnik GmbH) and 1.7% of *n*-octyl-β-d-glucopyranoside (β-OG; Anagrade, Affymetrix) was added, yielding a final bR concentration of about 0.9 mg ml^−1^ (as judged spectrophotometrically at 560 nm, absorption coefficient 63 000 l mol^−1^ cm^−1^). The suspension was sonicated in a bath sonicator for 1 min and incubated on a rock-roller. The next day, the pH was adjusted to 5.5 with 0.1 *M* HCl. The insoluble fraction was pelleted at 55 000 r min^−1^ (Ti 70 rotor; 15°C) for 45 min and the supernatant was concentrated in Amicon Ultra Centrifugal Filters (Ultracel-50k). The concentrated supernatant was applied onto a TSK G3000SW gel filtration column (TOSOH Bioscience) equilibrated with 1.2% β-OG in 25 m*M* NaH_2_PO_4_ pH 5.5. The bR from the first peak was discarded. The bR from the second peak was concentrated in Amicon Ultra Centrifugal Filters (Ultracel-50k) to a final concentration of 9 mg ml^−1^.

### 2.2. Crystallization   

The lipidic cubic phase (LCP) was prepared by mixing the protein sample with mono­olein (Nu-Check) in a 40:60 volume ratio using Hamilton syringes and a syringe coupler (Caffrey & Cherezov, 2009 ▶). Up to 20 µl of the LCP was injected into a 100 µl Hamilton syringe filled with precipitant solution composed of 29–38% polyethylene glycol 2000 (Fluka Analytical) and 100 m*M* Sorensen phosphate buffer pH 5.6 (KH_2_PO_4_ and Na_2_HPO_4_ from GERBU). A shower of small crystals appeared within a few days, with sizes varying with the batch of purified protein and the concentration of precipitant. All crystallization setups were prepared under dim red light and incubated at 20°C in the dark.

### 2.3. Sample preparation for LCP jet   

Before loading the samples into the LCP injector, the precipitant solution was removed from the syringe and mono­olein was added. To obtain a homogenous suspension of crystals in the LCP, samples were mixed through the syringe coupler. During this mixing step, larger crystals were broken into smaller fragments of less than 50 µm. The crystallization solutions were filtered and the protein samples were centrifuged to minimize the presence of dust particles in the final sample, as dust particles may block the LCP injector nozzle (the largest diameter used was 50 µm).

### 2.4. Data collection and processing   

**Figure 1:**
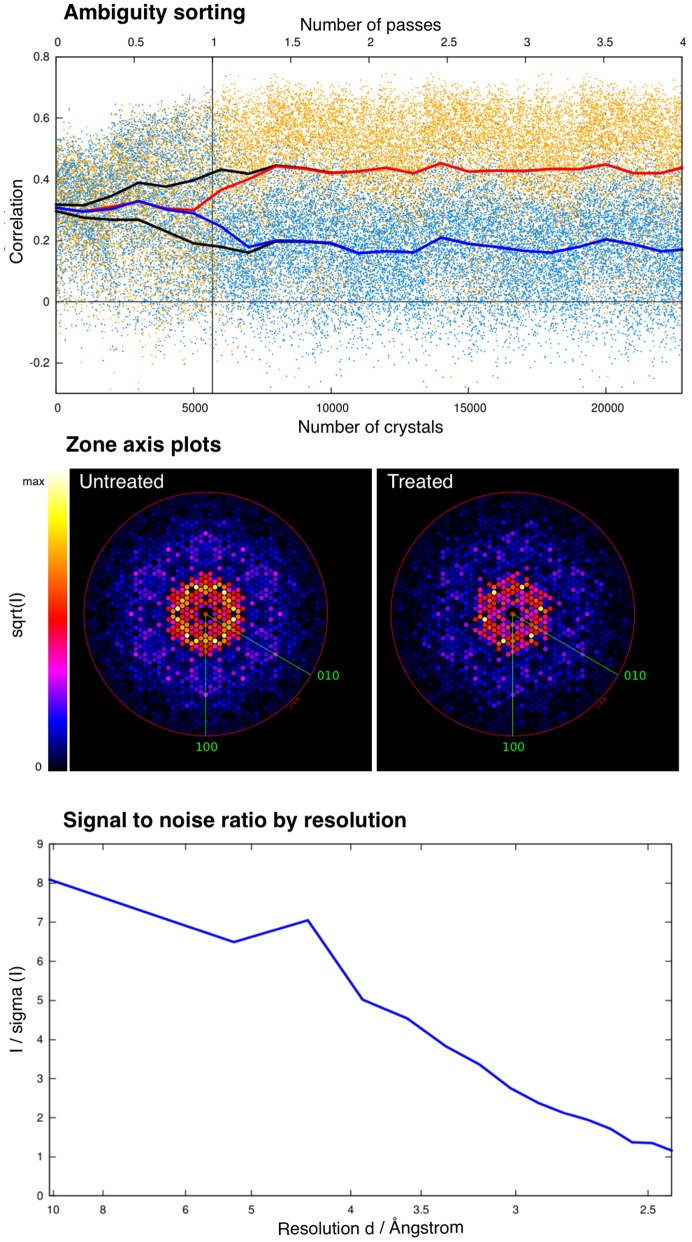
Data collection and refinement statistics for the SMX and CRYO bR structures. The upper inset shows the development of the correlation between the two indexing possibilities over the number of crystals used to resolve the indexing ambiguity. The middle inset shows zone-axis plots of the data before and after solving indexing ambiguity. Colours are proportional to the square root of the intensity (*i.e.* *I* ^1/2^). The lower inset plots the signal-to-noise ratio, expressed as *I*/σ(*I*), against the resolution of the SMX data.

Serial crystallographic data were collected at the ESRF Microfocus Beamline (ID13; Grenoble, France) in the setup described in the *Results* section. Alignment of the X-ray beam onto the LCP stream was facilitated with a grid scan of the area around the tip of the injector. Diffraction patterns were collected from randomly oriented crystals with 10–50 ms exposure times at a rate of 10–17 Hz. Due to random failures in the Rayonix MX-170 CCD detector, the upper left quadrant was excluded during data analysis. The detector was set to 4 × 4 binning mode with a pixel size of 177 µm and a frame size of 960 × 960 pixels. Because of overheads due to saving data on the ESRF data server, the frame rate was limited to 17 frames s^−1^. Collected images (details in Fig. 1 ▶) were pre-processed using *Cheetah* (Barty *et al.*, 2014 ▶) to exclude images without diffraction patterns. Higher hit rates and resolution were observed for crystals sized 20–40 µm. The *CrystFEL* program suite (White *et al.*, 2012 ▶) was used for data processing.

The conventional Cryo data set was collected at the PXI beamline of the Swiss Light Source (Paul Scherrer Institute, Villigen, Switzerland) in a cryostream at 100 K (details in Table. 1 ▶). The diffraction images were processed and scaled using *XDS* (Kabsch, 2010 ▶), followed by merging using *Aimless* (Evans & Murshudov, 2013 ▶).

### 2.5. Model building and refinement   

A single molecular replacement solution was found using the SMX data set and the structure of sensory rhodopsin II [PDB code 1h68 (Royant *et al.*, 2001 ▶), without ligands] as a search model in *Phaser* (McCoy *et al.*, 2007 ▶). Initial phases were used to rebuild the bacteriorhodopsin model automatically with *Phenix.autobuild* (Adams *et al.*, 2002 ▶), using simulated annealing refinement between iterative building steps. Manual building in *Coot* (Emsley & Cowtan, 2004 ▶) was used to complete the autobuild model, except for residues 1–4 and 234–249, and four side-chains for residues Q75, S158, K172 and R227. Further refinement was carried out using *PDBREDO* (Joosten *et al.*, 2012 ▶), which suggested one TLS group for the whole protein chain. In a final round of model building and refinement in *Refmac5* (Murshudov *et al.*, 2011 ▶), the model was completed with ten water molecules, five lipid fragments and all-*trans* retinal. The retinal was refined with restrained geometry of the Schiff base at the covalent link to Lys216.

The conventional Cryo data set was phased with *Phaser*, using bacteriorhodpsin [without ligands, PDB code 2ntu (Lanyi & Schobert, 2007 ▶)] as a search model. A single solution was found and the model was completed and refined using *Refmac5*. Retinal, 30 water molecules and eight lipid fragments were included in the last rounds of refinement. The unresolved region included the first four and last 17 residues, similar to the SMX structure. Moreover, a loop consisting of residues 157–163 was not included in the model obtained with the Cryo data, while for the SMX data it could be modelled into a weak electron density. Poorly resolved side-chains of K30, R164 and K172 were also not included in the model. The statistics are listed in Table. 1 ▶. The protein structures determined using the SMX and Cryo data sets have been deposited in the PDB with codes 4x31 and 4x32, respectively.

## 3. Results   

### 3.1. Experimental setup   

**Figure 2:**
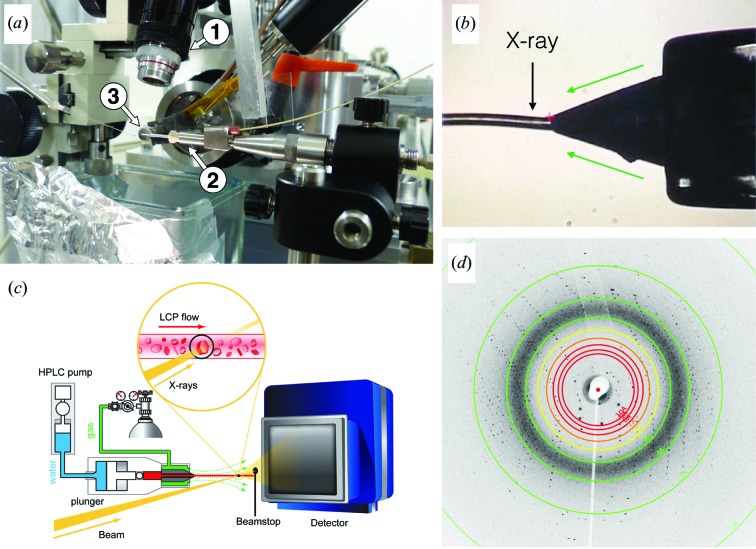
The experimental setup at the ID13 microfocus beamline. (*a*) (1) Microscope focused on the jet. (2) LCP injector with (3) nozzle close to the beamstop. (*b*) A view of the LCP nozzle as seen through the microscope. LCP was extruded towards the left as viewed in this projection, and the X-ray beam hits the stream at a distance of 40 µm from the end of the coned capillary. The capillary ID is 50 µm. A co-flowing gas stream (green arrows) keeps the LCP stream straight. (*c*) Schematic diagram of the setup. The water used to drive the injector is shown in blue, the LCP in red and the gas in green. (*d*) An SMX diffraction pattern from a bR microcrystal, with visible Bragg spots extending out to 2.2 Å resolution.

The LCP injector was installed horizontally at a 90° angle with respect to the X-ray beam (Fig. 2 ▶ *a*). A constant LCP flow of 20–60 nl min^−1^ through a 50 µm nozzle was stabilized by a co-axial flow of helium gas supplied at 7–10 bar (1 bar = 100 000 Pa). A video camera was used to monitor extrusion of the LCP column (Fig. 2 ▶ *b*). Several diffraction images from raster scans were used to align the 2 × 3 µm Gaussian-shaped X-ray beam onto the centre of the 50 µm wide LCP column, approximately 40 µm from the end of the injector nozzle. During data collection, a mechanical shutter interrupted the X-ray beam to collect diffraction images with exposure times of 10–50 ms (81% of images were collected at 25 ms) at a flux of up to 9.1 × 10^11^ photons s^−1^. The dead time between individual exposures was 55 ms which, combined with overheads related to the data-transfer rate, resulted in data acquisition at 10–17 Hz. At the chosen LCP flow rate, crystals moved 4–12 µm during a single exposure, continuously bringing fresh crystal sections or new crystals into the beam. In contrast with previous experiments at the LCLS, the LCP-SMX experiment described here is not performed in a vacuum environment, significantly reducing the complexity and cost of the experimental setup. Furthermore, LCP extrusion into air does not lead to a phase change in a monoolein-based LCP as observed in vacuum (Weierstall *et al.*, 2014 ▶), allowing collection of data at ambient temperature and pressure without the addition of special lipids.

### 3.2. Sample preparation and LCP injection   

Bacteriorhodopsin (bR) was the first protein for which the structure was solved (Pebay-Peyroula *et al.*, 1997 ▶) from crystals grown in the LCP (Landau & Rosenbusch, 1996 ▶), using data collected at the ID13 microfocus beamline. Since LCP-grown bR crystals diffract to high resolution and can be easily visualized due to their purple colour, bR is an ideal protein to demonstrate LCP-SMX at synchrotron sources. To produce a sufficient number of bR crystals for our experiment, we adapted previously published crystallization conditions (Nollert, 2004 ▶) to a setup using 100 µl gas-tight syringes (Supplementary Fig. S1), similar to that described elsewhere (Liu, Ishchenko & Cherezov, 2014 ▶; Liu, Wacker *et al.*, 2014 ▶). Once crystals had formed, excess crystallization buffer was removed and the residual buffer was absorbed by adding further monoolein. Manual operations, including loading of the injector, took only a few minutes. Less than 200 µg of protein was sufficient to fill the 20 µl reservoir of the LCP injector and collect data for 5–15 h, depending on the flow rate of the jet. To maximize the diffraction signal, the crystals should be as large as possible but still pass through the 50 µm capillary of the LCP injector. The concentration of crystals within the LCP also needs to be as high as possible in order to maximize the rate at which X-rays hit the crystals, but should optimally stay below the level at which multiple diffraction patterns are observed on a single diffraction image in order to simplify the analysis. Overall, we achieved a hit rate (number of diffraction patterns with >10 Bragg peaks/total number of images) of 0.5–2%, which is somewhat lower than the hit rates of 3–8% reported for similar experiments with different samples at the LCLS (Liu *et al.*, 2013 ▶; Weierstall *et al.*, 2014 ▶). This is probably due to the lower crystal density in our setup, as crystal size and crystal density were negatively correlated in our crystallization screening and we achieved the best diffraction with crystals of 20–40 µm, much larger than what would be ideal for data collection at an XFEL. Together with the lower data acquisition rate of 10–17 Hz at the ESRF compared with 120 Hz at the LCLS, the lower hit rate meant that the collection of this data set took 3 d. Nevertheless, only 0.8 mg of protein in 200 µl of LCP was needed.

Occasionally, two or three consecutive hits were recorded on some of the larger (40–50 µm) crystals. Bragg spots appeared and disappeared within this sequence of consecutive images, indicating that the rotational diffusion of crystals in the LCP within the 80 ms between two exposures is larger than their mosaic spread. To investigate this further, a computer program was written to compare the orientations of crystals in adjacent frames using the data stream output from *CrystFEL*. For the fraction of data acquired with a 25 ms exposure time (81% of the total frames), 1088 frames (26% of the successfully indexed patterns) were found to be part of a rotation series. The mean series length was 2.2 frames and the maximum series length was 4 frames. Such a series of consecutive diffraction patterns might be useful for indexing and integration, as it resembles a small wedge of rotation data similar to those typically collected in conventional crystallography. A further reduction in the LCP flow rate and an increase in the frame rate could thus be used to collect more images from the same crystal and increase the overall data collection efficiency.

### 3.3. Data processing and map calculation   

We collected 1 343 092 images, of which 12 982 were classified as hits using the *Cheetah* program (Barty *et al.*, 2014 ▶), giving an average hit rate of ∼1%. A large fraction of the frames were found to exhibit artifacts in one quadrant of the detector, and this quadrant was therefore ignored for all stages of analysis. The unit-cell parameters were determined to be *a* = *b* = 62.79 Å and *c* = 109.67 Å in space group *P*6_3_ during initial indexing of a subset of the data, consistent with the known lattice of bR crystallized in LCP. Of the initial hits, 5691 images were successfully indexed and integrated by *CrystFEL* (Version 0.5.3a+e2c7dbd5) without difficulty. However, the space group of bR crystals is subject to an indexing ambiguity [see White *et al.* (2013 ▶) for an extensive discussion], which was resolved by *CrystFEL* using an algorithm related to one recently developed for this purpose [Brehm & Diederichs (2014 ▶); see Liu & Spence (2014 ▶) for a solution based on an expectation maximization algorithm], prior to merging the individual intensity measurements for each symmetrically unique reflection according to point group 6/*m* (*i.e.* Friedel pairs were also merged). The resolution limit of the diffraction signal in the merged intensities was judged as 2.4 Å, based on signal-to-noise ratios, CC*, visual inspection of the density (Supplementary Fig. S2) and suggestions by the *PDB Redo* web server (Joosten *et al.*, 2012 ▶).

For comparison against this SMX data, we harvested a single bR crystal of ∼50 × 50 × 10 µm and collected data at the Swiss Light Source under cryogenic conditions. This conventionally collected data set (Cryo) has a resolution of 1.9 Å with no detectable signs of twinning (as determined by *Phenix.xtriage*; Adams *et al.*, 2002 ▶). We confirmed this finding using several other single crystals, as all previously described bR crystals grown in LCP show various degrees of twinning (Wickstrand *et al.*, 2014 ▶). It is possible that this improvement in crystal quality is due to a change in crystallization conditions, since we used polyethylene glycol as precipitant to avoid high concentrations of salts for future XFEL experiments. A comparison of the two data sets is shown in Fig. 1 ▶.

An important consideration for every structure determined by molecular replacement is the potential impact of model bias, which may hide differences between the model used for structure determination and the true structure. To limit the impact of model bias, we used the related sensory protein rhodopsin II [PDB code 1h6b (Royant *et al.*, 2001 ▶), ∼30% sequence identity with bR] for molecular replacement with the SMX data (supplementary Fig. S3) and subjected the solution to automatic model building using simulated annealing refinement in *Phenix* (Adams *et al.*, 2002 ▶). Manual addition of lipid fragments, water molecules and the retinal co-factor, combined with a final round of refinement, resulted in a bR structure that is essentially free of model bias, providing a clear demonstration of the quality of our SMX data.

## 4. Comparison of Cryo and SMX structures   

**Figure 3:**
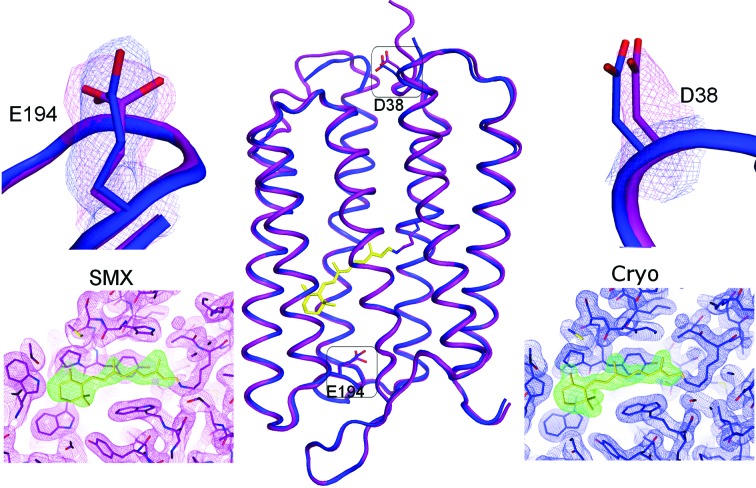
(Centre) Comparison of bR structures solved by SMX and conventional cryocrystallography (Cryo). The protein backbone of the room-temperature SMX structure (purple) superimposes well with the Cryo structure (blue). (Bottom) Retinal omit maps [blue (Cryo) or purple (SMX) mesh, 2*F*_o_ − *F*_c_ at 1.5σ; green mesh, *F*_o_ − *F*_c_ at 2.5σ] indicate increased flexibility in the β-ionone ring. The upper insets show a different rotamer for E194 involved in proton translocation, and indications for radiation damage on D38 exposed to the extramembrane environment.

**Figure 4:**
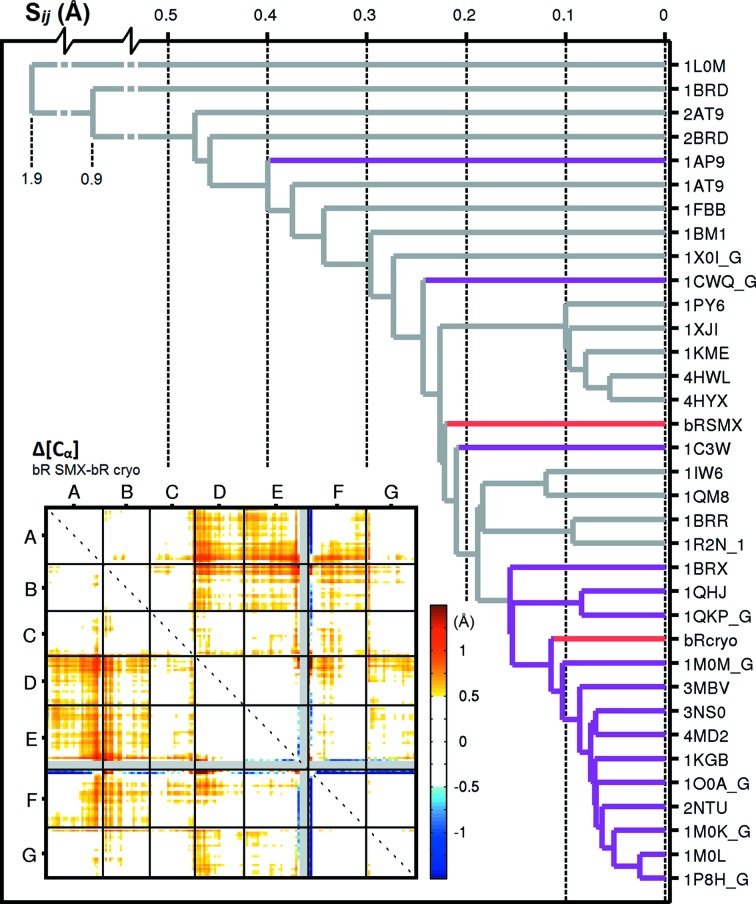
Cluster analysis of bR ground-state structures using hierarchical sorting. This analysis sorts according to the average of the absolute value of the difference between two internal distance matrices [*S*_*jj*_(Å)] calculated on C^α^ atoms (Wickstrand *et al.*, 2014 ▶). PDB codes are given for all deposited wild-type structures of bR in its resting state. LCP bR structures are marked in purple. The room-temperature SMX structure (bRSMX) and the structure of bR solved here using conventional data collection and cryocooling (bRcryo) are marked in red. Inset: The internal distance matrix for bRSMX–bRcryo shows that cryocooling compresses helices A and B slightly towards helices D, E and F.

The synchrotron cryocooled bR structure was solved using molecular replacement with ground-state bR (PDB code 2ntu; Lanyi & Schobert, 2007 ▶). Overall, this structure and the bR structure collected by SMX at room temperature are very similar (Fig. 3 ▶), with a C^α^ root mean-square deviation of 0.54 Å. The distribution of crystallographic *B* factors is comparable between the two structures, but the *B* factors are slightly higher in the SMX bR structure, probably because of the lower resolution or increased thermal motion of the protein at room temperature. We observed weak electron density for the loop between helix E and helix F, and were thus able to include this loop in the SMX bR structure despite the lower resolution. Hierarchical cluster analysis (Wickstrand *et al.*, 2014 ▶) reveals average differences in the internal distance matrix (*S*_*ij*_) of the order of 0.2 Å relative to most published structures of the bR ground state (Fig. 4 ▶). This difference is primarily due to a small perturbation of helices D, E and F away from helices A and B relative to the structure solved with the Cryo data. This effect potentially reflects an impact of cryocooling, compressing the bR structure slightly, rather than differences in data collection, since it is well known that cryocooling can introduce small structural perturbations. Analysis of data from 30 proteins (Fraser *et al.*, 2011 ▶), for example, indicated that cooling changes the side-chain conformations for 35% of surface-exposed residues. Comparison of serotonin receptor 2B (5-HT2B) crystal structures collected using microcrystallography under cryoconditions (Wacker *et al.*, 2013 ▶) and by LCP-SFX (Liu *et al.*, 2013 ▶) revealed similar changes in surface residues. At a more detailed level, structural comparison of the SMX and Cryo data sets reveals rotamer changes in a series of amino acids on the bR surface, but also in internal residues like Glu194 (Fig. 3 ▶, upper left panel). The ligand-binding pocket is identical in the SMX and Cryo structures (Fig. 3 ▶, lower insets). In each case, the retinal ligand is well resolved and, when omitted during refinement, clear positive density emerges in the resulting difference maps, *mF*_o_ − *DF*_c_. In both cases, retinal is co­valently bound to Lys216 and in the all-*trans* conformation, as expected for the bR ground state. The extent to which the minor structural differences observed here are physiologically relevant is presently unclear.

## 5. Discussion   

Several crystal structures have demonstrated the potential of the LCP injector for SFX of membrane proteins using XFELs (Liu *et al.*, 2013 ▶; Caffrey *et al.*, 2014 ▶; Weierstall *et al.*, 2014 ▶). In this study, we have adapted the technology for use at more widely available synchrotron-based microfocus beamlines and have demonstrated that room-temperature LCP-SMX of membrane proteins is possible at synchrotron sources.

One of the problems that had to be overcome was the indexing ambiguity, with data collected from multiple crystals with merohedral point groups. Even though 27 out of 65 space groups may suffer from indexing ambiguities, it rarely causes problems in conventional crystallography, as all indexing regimes are equally valid and only one has to be selected for an individual large single crystal. Even in the case when data from multiple crystals need to be merged, data sets are likely to have a large enough overlap on which to base common indexing. Single diffraction snapshots taken in serial crystallography provide only a set of partial reflection intensities, for which there are two indexing possibilities in the present case (space group *P*6_3_). Direct merging of these images without regard to intensities leads to the formation of a perfectly twinned data set of higher symmetry (with equal contributions from each indexing mode) that is very prone to model bias and poorly suited for structure determination and refinement. Here, we have provided an example of how indexing ambiguity can be resolved, and demonstrate that serial crystallography is not limited to non-merohedral space groups.

Today, around 95% of protein structures are determined from cryocooled crystals. While in our case the structural differences between room-temperature and cryogenic data collection were small, in some cases cryogenic cooling can change the dynamic behaviour of proteins and may lead to structural artifacts (Fraser *et al.*, 2011 ▶; Keedy *et al.*, 2014 ▶). The reason why cryogenic data collection is still so dominant is that cryocooling reduces radiation damage, which is the major factor limiting the amount and quality of structural information that can be obtained from a protein crystal (Garman, 2010*b* ▶). Primary radiation damage is a result of the X-ray photo­ionization of atoms in the crystal or surrounding liquor, and the subsequent rapid cascade of electron collisional ionization that takes place in several hundred femtoseconds, resulting in low-energy solvated electrons and hydroxyl radicals. Secondary damage refers to the radiochemistry due to these radicals, which are able to diffuse and react with particular components such as metal centres and disulfide bridges, and lead to decarboxylation of aspartate and glutamate residues (Allan *et al.*, 2013 ▶; Davis *et al.*, 2013 ▶). Tertiary damage is defined as the effect on the crystal lattice and other mechanical consequences of the energy deposition in the crystal.

A new method to limit radiation damage is to outrun its consequences and finish the measurement before the damage causes significant loss of information at the resolution of interest (Neutze *et al.*, 2000 ▶). Even with an exposure time of 100 ms, 50% of global radiation damage can be avoided at near room temperature by outrunning secondary and tertiary damage effects (Owen *et al.*, 2012 ▶; Warkentin *et al.*, 2013 ▶). The effect is already exploited by *in situ* diffraction methods, where the crystals are directly exposed in crystallization plates and diffraction images from dozens of crystals are merged to give a complete data set (Axford *et al.*, 2012 ▶). In a similar approach, X-ray semitransparent microfluidic chips have been used to collect and merge partial rotation series from multiple room-temperature crystals (Khvostichenko *et al.*, 2014 ▶). At the Swiss Light Source, *in situ* screening has been very successful as part of an integrated crystallization and diffraction screening platform (Bingel-Erlenmeyer *et al.*, 2011 ▶). So far, such *in situ* diffraction techniques have not yet been developed into a routine method for data collection, mainly due to the modifications needed at synchrotron beamlines, rotational limitations, background from conventional crystallization plates and radiation damage. The LCP injector used in our SMX experiment provides a constant stream of fresh crystals, so that each of the several thousand crystals contributing to the final data set is exposed for only 10–50 ms. The total radiation dose to the exposed region of a single crystal is thus only about 0.7 MGy, compared with the 28 MGy deposited during collection of the conventional data set (calculated using *RADDOSE-3D*; Zeldin *et al.*, 2013 ▶). Per crystal, the dose thus remains below the Henderson–Garman safe dose limit of 1 MGy at room temperature and 30 MGy for frozen samples (Garman & McSweeney, 2007 ▶). Indeed, we could not detect radiation damage by investigation of radiation-sensitive residues in the SMX structure, although it has been argued that very subtle shifts near the retinal Schiff base can be observed in high-resolution cryo structures of bR at a dose as low as 0.06 MGy (Borshchevskiy *et al.*, 2014 ▶). By contrast, the structure obtained from cryocooled bR crystals (28 MGy dose) showed weak density for several aspartate residues (Fig. 3 ▶, upper right inset), an indication of mild radiation damage that could possibly be avoided by further truncation of the data. Nevertheless, this comparison shows that serial injection of crystals at a synchrotron using an LCP injector allows collection of data at room temperature with minimal radiation damage.

A further potential application for LCP-SMX at room temperature is time-resolved crystallography. Cryocooled crystals have been used extensively for low-temperature trapping of bR intermediates [reviewed by Wickstrand *et al.* (2014 ▶)] but cannot be used for genuinely time-resolved pump-probe experiments. Room-temperature LCP-SMX can be adapted for time-resolved diffraction studies by using laser pulses to photoactivate the microcrystals and varying the time of arrival of the photoactivating laser pulse relative to when the X-ray image is recorded. By using rapid readout X-ray detectors, it is already possible to achieve millisecond time resolution. The use of polychromatic X-ray beams in combination with microfocusing should allow time-resolved SMX to be extended to timescales as short as 100 ps in favourable cases (Schotte *et al.*, 2003 ▶). Resolving dynamics on timescales much faster than this will remain dependent on the femto­second pulses of XFELs (Arnlund *et al.*, 2014 ▶).

A common problem with LCP is its high viscosity, which makes crystal harvesting a somewhat difficult procedure, especially for inexperienced users. In addition, crystals are often invisible through the beamline camera, owing to the opacity of the cryocooled lipidic mesophase surrounding them. This makes alignment with the X-ray beam a time-consuming process for which specific techniques like X-ray beam rastering (Cherezov *et al.*, 2009 ▶) or X-ray imaging (Warren *et al.*, 2013 ▶) are required. Serial injection of microcrystals by LCP-SMX, as demonstrated here, has the potential to simplify this process by eliminating pre-exposure, handling and centring of crystals. Improvements in sample preparation, as well as the use of next-generation single-photon counting X-ray pixel detectors with a higher frame rate beyond 1 kHz frequency and a shorter readout dead time in the microsecond range, will allow matching of the rate at which crystals traverse the beam with the desired exposure time, resulting in more efficient data collection. The practically zero readout noise of modern detectors compared with the CCD detector we were limited to in our experiment will further increase the achievable resolution. Another important factor is background scattering from the stream of LCP. It is most prevalent in a diffuse ring around 4.5 Å resolution and less compromising for lower and higher resolution ranges. Data collection using nozzles with a smaller diameter will further decrease background scattering and increase the quality of collected data, but such nozzles have to be chosen carefully according to the crystal size so as to not block the injector. Judicious choice of flow rate, jet diameter, crystal size and exposure time may also allow sufficient microcrystal rotation during an exposure to generate fuller reflections. Future upgrades to modern synchrotron sources will increase the available flux by several orders of magnitude and further reduce exposure times and the size of crystals that can be measured. With these improvements, the method could be particularly useful for the investigation of pharmacologically relevant human proteins that are often expressed in only small quantities. The simplified crystal handling, compared with conventional crystal harvesting and cryofreezing, should be well suited for automation and high-throughput approaches. We also foresee synergies for synchrotron-based SMX and XFEL-based SFX, as these complementary approaches are used to accelerate the pace of discovery for the most challenging classes of proteins in structural biology.

## Supplementary Material

PDB reference: bacteriorhodopsin using SMX, 4x31

PDB reference: bacteriorhodopsin using conventional crystallography, 4x32

Supporting figures. DOI: 10.1107/S2052252514026487/jt5008sup1.pdf

## Figures and Tables

**Table 1:** Data-collection and refinement statistics for SMX bR and cryo bR structures.

	SMX	Cryo
Data collection		
X-ray source	ID13, ESRF	PXI-X06SA, SLS
Detector	Rayonix MX-170 CCD	PILATUS 6M
Temperature (K)	294	100
Wavelength ()	0.954	1.000
Beam size (m)	2 3	50 10
Average crystal size (m)	540 540 15	50 50 10
Flux (photonss^1^)	9.1 10^11^	5.9 10^11^
Space group	*P*6_3_	*P*6_3_
Unit-cell parameters (, )	*a* = *b* = 62.8, *c* = 109.7, = = 90, = 120	*a* = *b* = 60.5, *c* =101.5, = = 90, = 120
Oscillation ()/exposure (ms)	n.a./1050 (81% 25)	0.1/150
No. of collected images	1343092	2532
No. of hits/indexed images	12982/5691	2532/2532
Total/unique reflections	1223766/9655	234541/16643
Resolution range ()	36.562.40 (2.462.40)	46.571.90 (1.941.90)
Completeness (%)	100.0 (100.0)	100.0 (100.0)
Multiplicity	127 (88.8)	14.1 (14.3)
*I*/(*I*)	3.57 (1.16)	17.90 (1.80)
CC^*^ †	0.981 (0.658)	1.000 (0.841)
*R*_split_ ‡ (SMX) or *R*_p.i.m._ (cryo) (%)	22.4 (107)	2.6 (50)
Matthews coefficient* V*_M_ (^3^ Da^1^)	2.50	2.21
Solvent content (%)	50.76	44.27
*B* factor from Wilson plot (^2^)	45.2	33.4
Refinement		
Resolution range ()	31.402.40 (2.462.40)	52.421.90 (1.951.90)
No. of reflections (total/test set)	9192/441	15773/841
*R*_work_/*R*_free_ (%)	20.5/24.9	17.1/21.4
No. of atoms		
Overall	1848	1877
Protein	1756	1723
Retinal	20	20
Water	10	30
Lipids and other	62	104
Average *B* factors (^2^)		
Overall	40.47	28.50
Protein	39.04	27.11
Retinal	52.67	24.61
Water	55.94	36.52
Lipids and other	74.47	49.93
R.m.s. deviations		
Bond lengths ()	0.008	0.009
Bond angles ()	1.01	1.21
Ramachandran favoured (%)	98.2	98.9
Ramachandran outliers (%)	0.4	0.0

† CC* = [2CC_1/2_/(1 + CC_1/2_)]^1/2^.

‡ *R*_split_ = 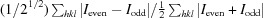.
