# Supplementary material for: Lipidic cubic phase serial millisecond crystallography using synchrotron radiation
Source: IUCrJ. 2015 Jan 27;2(Pt 2):168–76. doi: 10.1107/S2052252514026487 (PMC4392771; doi:10.1107/S2052252514026487)
Supplement: Supplementary file 1 [file m-02-00168-sup1.pdf]

IUCrJ

Volume 2 (2015)

Supporting information for article:

Lipidic cubic phase serial millisecond crystallography using synchrotron radiation

Przemyslaw Nogly, Daniel James, Dingjie Wang, Thomas A. White, Nadia Zatsepin, Anastasya Shilova, Garret Nelson, Haiguang Liu, Linda Johansson, Michael Heymann, Kathrin Jaeger, Markus Metz, Cecilia Wickstrand, Wenting Wu, Petra Bath, Peter Berntsen, Dominik Oberthuer, Valerie Panneels, Vadim Cherezov, Henry Chapman, Gebhard Schertler, Richard Neutze, John Spence, Isabel Moraes, Manfred Burghammer, Joerg Standfuss and Uwe Weierstall

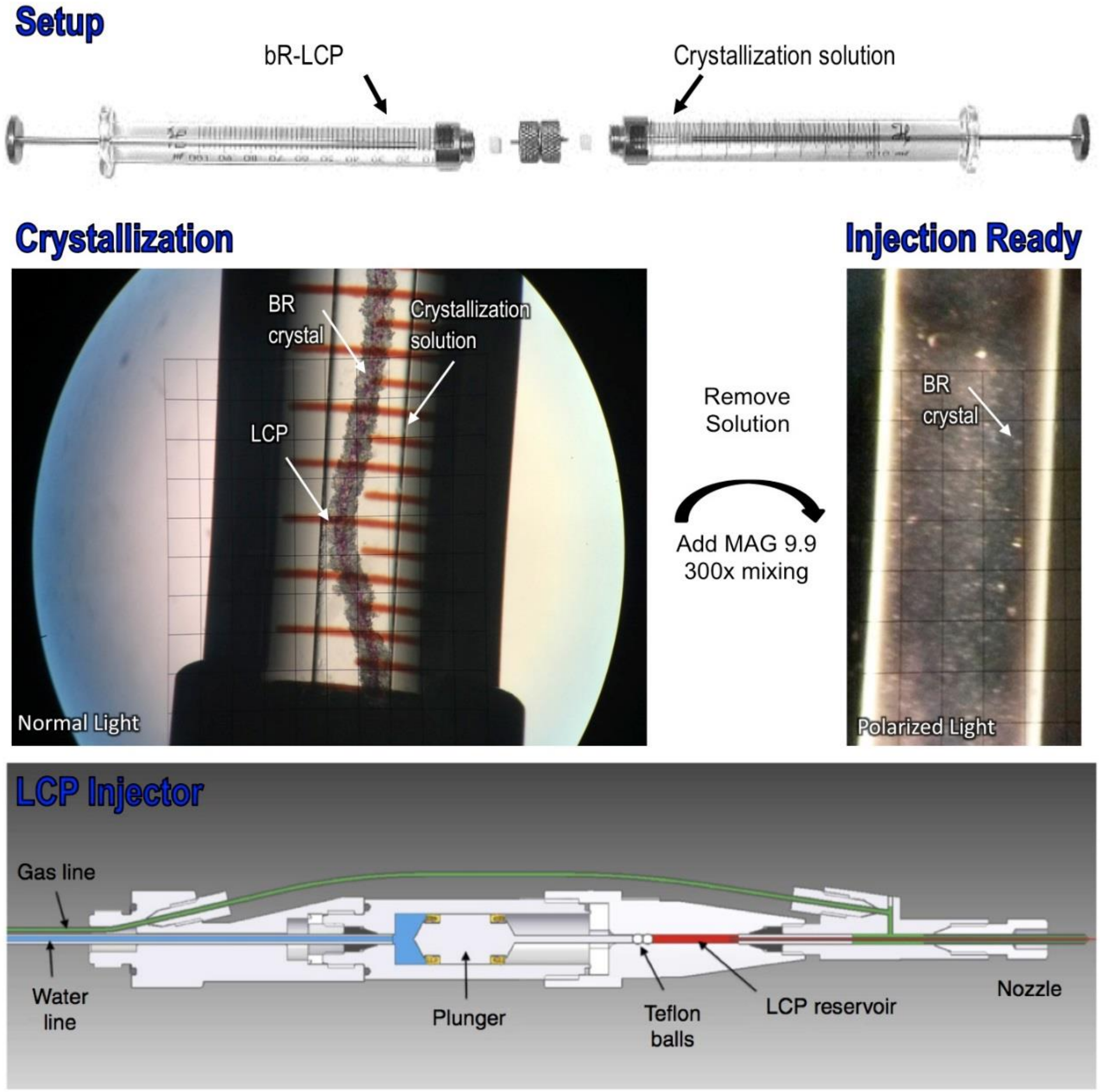

Figure S1 Preparation of bR crystals in gas-tight syringes for LCP-SMX experiments. Upper panel showing a set of 100 µl Hamilton syringes as used in the crystallization setup. Middle panel shows bR crystals grown in the LCP tube immersed in precipitant (left) and crystals sample after removing the precipitant (right). Below is a schematic drawing of the LCP micro extrusion device used for sample injection (Weierstall *et al.*, 2014).

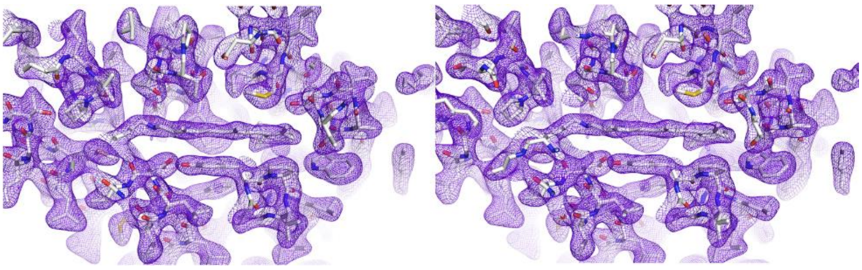

Figure S2 Stereoview of the bR SMX electron density map in the region of retinal binding pocket (2Fo-Fc at 1.0 sigma level).

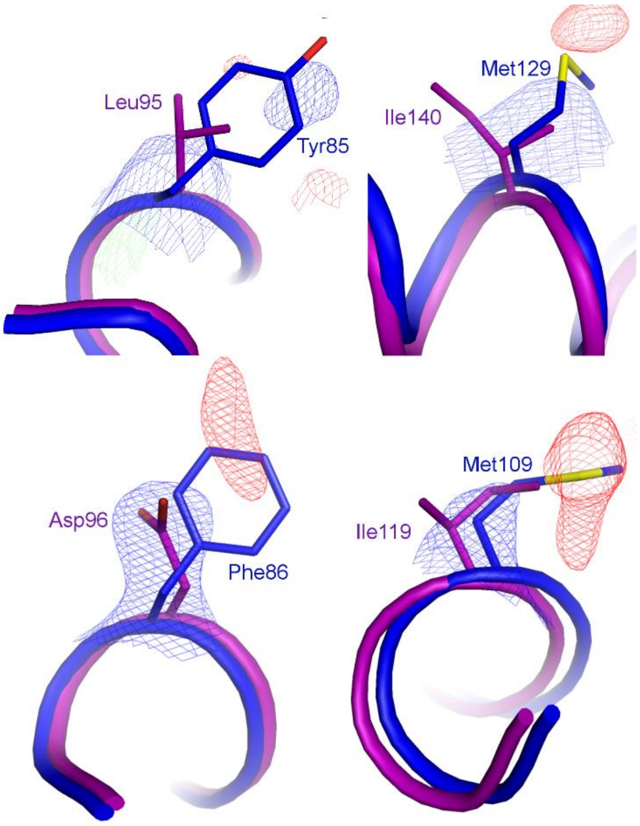

Figure S3 Electron density observed for SMX data after molecular replacement with Sensory rhodopsin II (blue) and the corresponding residues of bR (PDB code 2NTU, purple). Blue mesh represents 2Fo-Fc density at 1.0 sigma. Green and red meshes correspond to the Fo-Fc map shown at 3 sigma. Already in these initial maps many side chains that differ between sensory rhodopsin II and bR can be assigned. The ability to complete bR by automated model building routines using these maps clearly demonstrates the quality of the collected SMX data.
